# Supplementary material for: Structural basis of DNA packaging by a ring-type ATPase from an archetypal viral system
Source: Nucleic Acids Res. 2022 Aug 10;50(15):8719–32. doi: 10.1093/nar/gkac647 (PMC9410871; doi:10.1093/nar/gkac647)
Supplement: gkac647_Supplemental_File [file gkac647_supplemental_file.pdf]

Supplementary Material for

Structural basis of DNA packaging by a ring-type ATPase from an archetypal viral system

Herman K.H. Fung, Shelley Grimes, Alexis Huet, Robert L. Duda, Maria Chechik, Joseph Gault, Carol V. Robinson, Roger W. Hendrix, Paul J. Jardine, James F. Conway, Christoph G. Baumann*, Alfred A. Antson*

* To whom correspondence should be addressed. Tel: (44) 1904 328255, (44) 1904 328828; Email: fred.antson@york.ac.uk, christoph.baumann@york.ac.uk

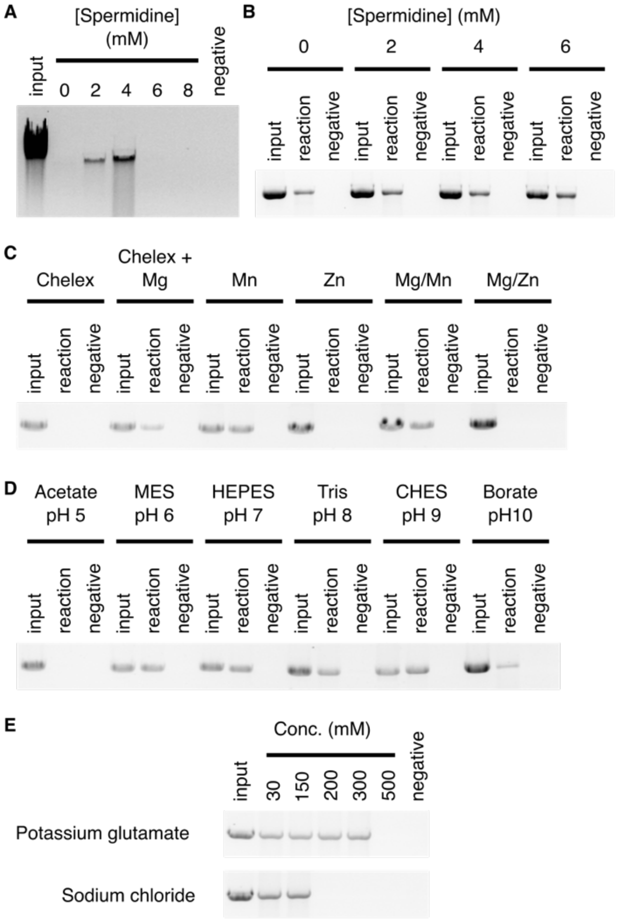

Figure S1. Biochemical properties of the HK97 DNA packaging system. Dependence on spermidine in the packaging of (**A**) the HK97 genome and (**B**) linear *cos*-containing pUC18 DNA. Dependence on (**C**) divalent metal ions, (**D**) pH and (**E**) monovalent salts in the packaging of linear cos-containing pUC18 DNA. Reaction components were treated with Chelex-100 resin before addition of divalent metal ions. Samples with different salt levels were ethanol-precipitated before analysis by agarose gel electrophoresis. Ethidium bromide staining was used to visualize DNA in the agarose gel.

Table S1. TerL crystallographic data collection and refinement statistics.

	SeMet *peak*	*inflection*	KBr soak
**Data collection**			
Wavelength (Å)	0.97949	0.97969	0.91983
Space group	*C*2	*C*2	*C*2
Cell dimensions			
a, *b*, *c* (Å)	211.5, 39.3, 66.0	211.9, 39.3, 66.1	211.8, 39.2, 65.5
β (°)	103.3	103.2	103.4
Resolution (Å)	49.59–2.00 (2.05–2.00)	49.72–2.40 (2.49–2.40)	45.53–2.20 (2.27–2.20)
R_{merge}	0.122(1.233)	0.100 (1.249)	0.136 (0.842)
$I / \sigma(I)$	10.1 (1.0)	13.6 (1.6)	7.1 (1.8)
$CC_{1/2}$ (%)	99.7 (36.5)	99.9 (52.9)	99.1 (59.5)
Completeness (%)	98.3 (85.3)	99.9 (99.7)	99.6 (98.2)
Redundancy	6.2 (4.1)	6.6 (6.4)	3.3 (3.3)
Wilson B (Å^2)	43.3	60.5	37.6
No. unique reflections	35605 (2257)	21206 (2198)	26796 (2242)
**Refinement**			
Resolution (Å)			45.53–2.20
No. reflections			
Working			25551
Free			1244
R_{work}/R_{free}			0.205/0.254
No. atoms			
Protein			3522
Water			93
Ligand			18
B factors			
Protein			38.9
Water			33.3
Ligand			53.1
R.m.s. deviations			
Bond lengths (Å)			0.008
Bond angles (º)			1.3
Ramachandran			
Favored (%)			97.5
Outlier (%)			0.0

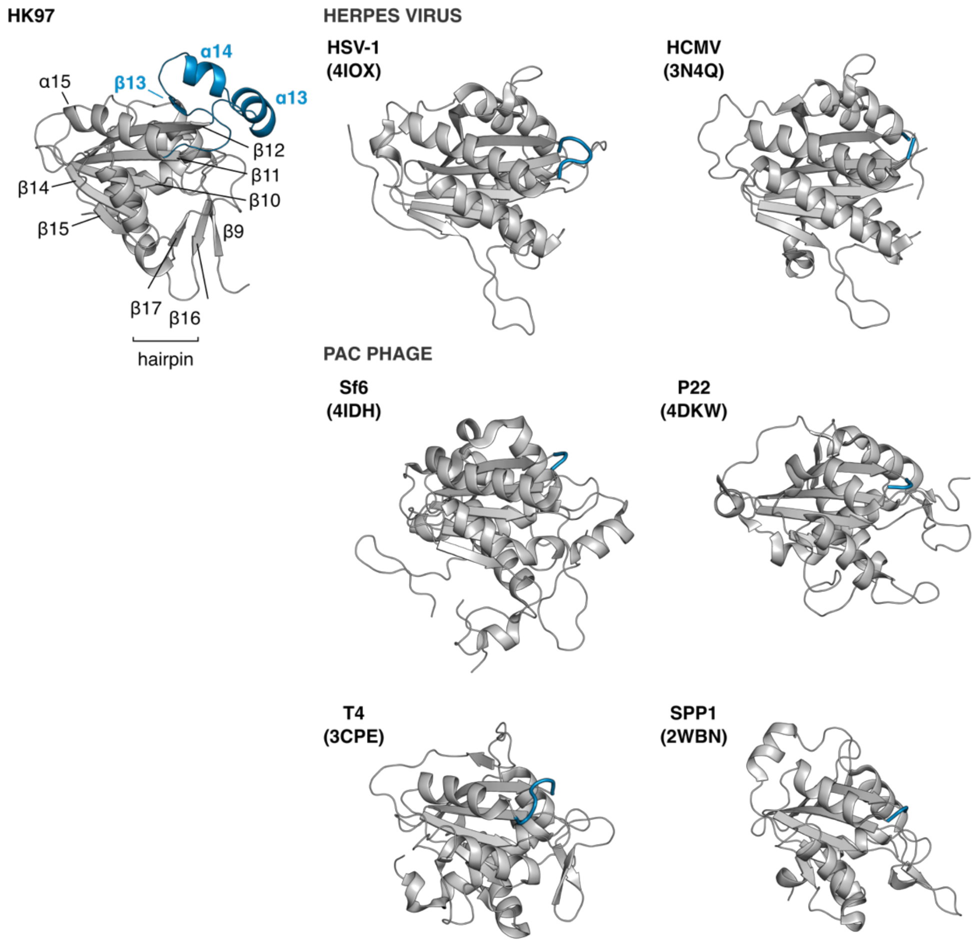

Figure S2. Comparison of viral packaging nucleases. The HK97 TerL nuclease contains an additional ααβ element, colored blue, on the periphery of the central β-sheet. The corresponding region in herpes virus and *pac* homologues is colored blue. PDB codes are indicated in brackets.

A

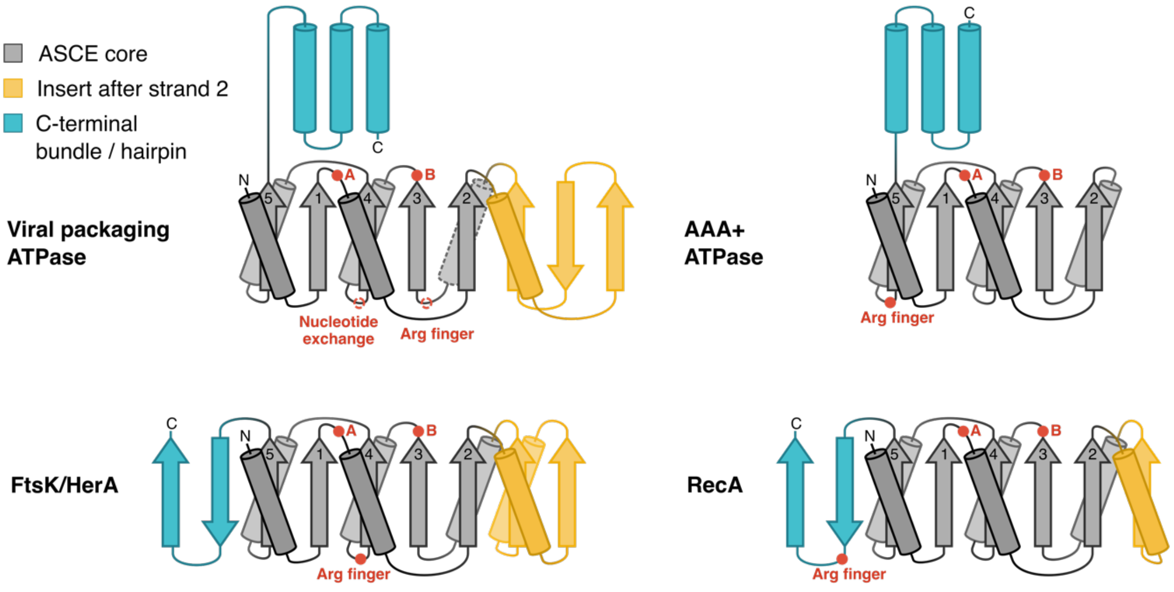

B

		Walker A	
**HK97_GP2**	50	AILS IARKNGKT GLIAGIL	68
**HSV1_UL15**	254	TVFL VPRRHGKT WFLVPLI	272
**HCMV_UL89**	208	TVFL VPRRHGKT WFIIPII	226
**HSV2_UL15**	254	TVFL VPRRHGKT WFLVPLI	272
**HHV8_AAB08384**	217	SVFL IPRRHGKT WIVVAII	235
**T4_GP17**	156	TVCN LSRQLGKT TVVAIFL	174
**P74-26_YP_001468054**	33	RVAC LGRQSGKS EAASVEA	51
**T7_GP19**	54	FILQ AFRGIGKS FITCAFV	72
**T5_AAU05290**	57	VTAC VSRRVGKS FIAYTLG	75
**SPP1_GP2**	29	YVLK GGRGSAKS THIAMWI	47
**Sf6_GP2**	18	KVAK GGRGSGKS WAIARLL	36
**Phi29_GP16**	20	NFVI GARGIGKS YAMKVYP	38
**P22_GP2**	56	RCFM AGNQLGKS FTGAAEV	74
**Lambda_GPA**	72	VNVV **K**SARVGYS **K**MLLGVY	90

Figure S3. Comparison of ASCE ATPase topologies and Walker A sequences. (**A**) Topology diagrams of select ASCE (additional strand conserved E) ATPase subfamilies. AAA+, ATPases associated with diverse cellular activities. The consensus fold for viral packaging ATPases across all structurally characterized *cos*, *pac* and phi29 systems are shown. The gray helix in the ASCE core with a dotted outline is a random coil in the HK97 TerL. The phi29 ATPase has an additional β-strand between ASCE strand 5 and preceding helix (not shown). The location of confirmed *trans*-acting arginine fingers are indicated for each ATPase subfamily. (**B**) Multiple sequence alignment of viral packaging ATPase Walker A sequences. The virus and gene product names are indicated, separated by an underscore. In grey is the classical Walker A lysine; red, critical arginine residue identified in *pac* phage T4, *cos* phage lambda and phi29; yellow, critical lysine residue in HK97 and corresponding positive residues in phage T5 and herpes viruses. Lambda gpA has an unusual Walker A sequence with two lysine residues upstream and downstream of the classical position (in bold), both required for ATPase and DNA packaging activities (1, 2).

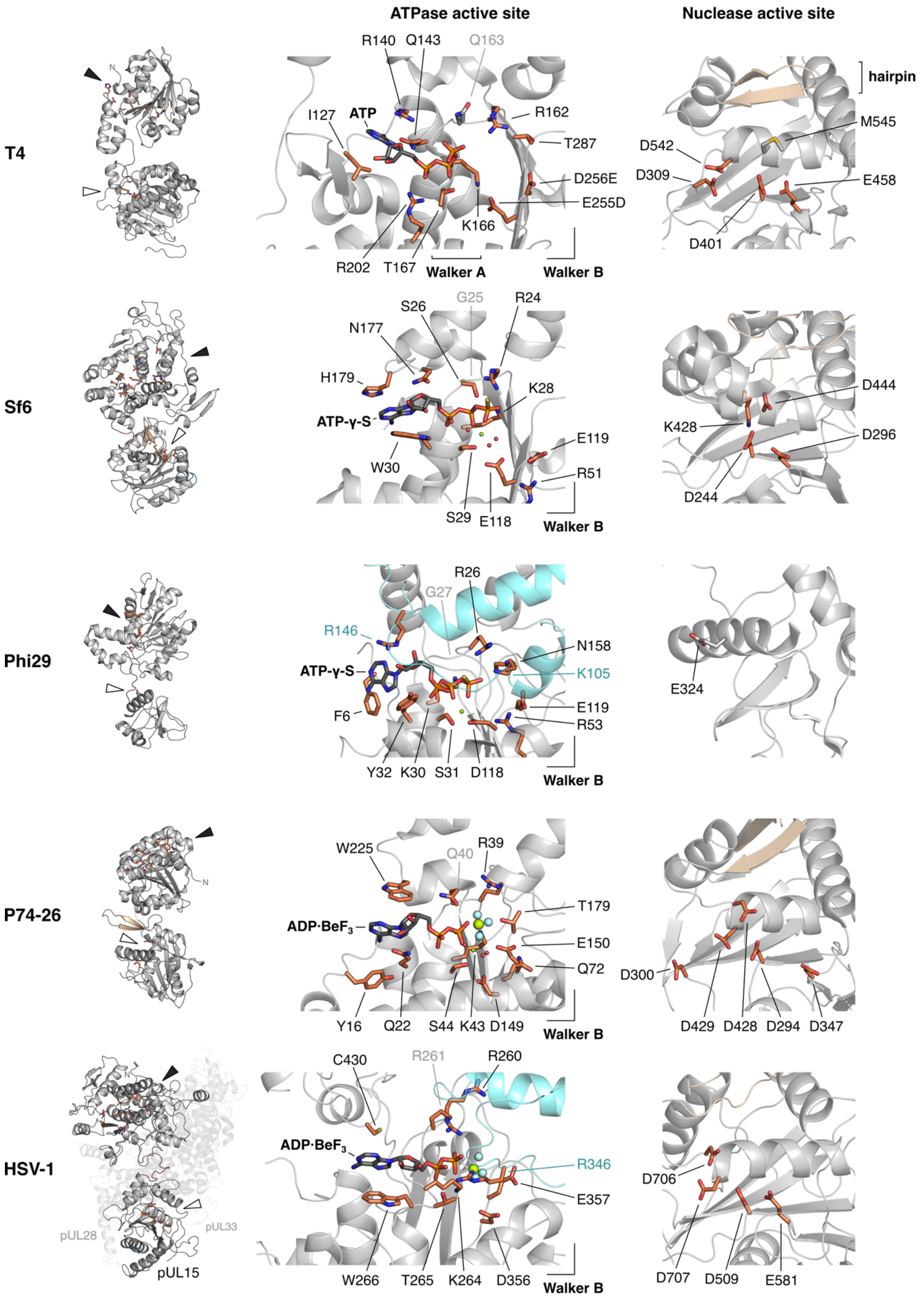

ATPase active site
Nuclease active site
T4
R140
Q143
Q163
I127
ATP
R162
T287
D256E
E255D
K166
R202
T167
Walker A
Walker B
hairpin
M545
D542
D309
E458
D401
Sf6
S26
G25
R24
N177
H179
K28
ATP-γ-S
E119
W30
R51
S29
E118
Walker B
D444
K428
D296
D244
Phi29
R26
G27
R146
N158
ATP-γ-S
K105
E119
F6
R53
Y32
K30
S31
D118
Walker B
E324
P74-26
W225
Q40
R39
T179
ADP·BeF3
E150
Q72
Y16
Q22
S44
K43
D149
Walker B
D300
D429
D428
D294
D347
HSV-1
C430
R261
R260
ADP·BeF3
R346
E357
W266
T265
K264
D356
Walker B
pUL28
pUL33
pUL15
D706
D707
D509
E581

Figure S4. Comparison of TerL active sites. Left, full-length structures of *pac* phage T4 and Sf6 TerL, phi29 gp16 (PDB 3CPE, 4IDH, 7JQQ), the ATPase and nuclease domains of *pac* phage P74-26 TerL (PDB 4ZNL, 5TGE), and the tripartite monomer of the herpes simplex virus type 1 (HSV-1) hexameric terminase assembly (PDB 6M5V). The ATPase and nuclease active sites are indicated with a black and white arrowhead, respectively. Active site residues are shown as sticks. Secondary structure elements corresponding to the HK97 protein are colored as in Figure 2 (red, blue, beige). Middle, the ATPase active site. Structures in complex with ATP, ATP-γ-S or ADP·BeF_3 are shown (PDB 2O0H, 4IEE, 7JQQ, 4ZNL, 6M5V). *Trans*-acting elements from an adjacent subunit are shown in blue, where available. Grey-labelled residues correspond to the unusual lysine K57 of HK97. Right, the nuclease active site. Putative or confirmed residues important for divalent metal ion coordination for DNA cleavage are shown. The phi29 vestigial nuclease domain is shown for comparison.

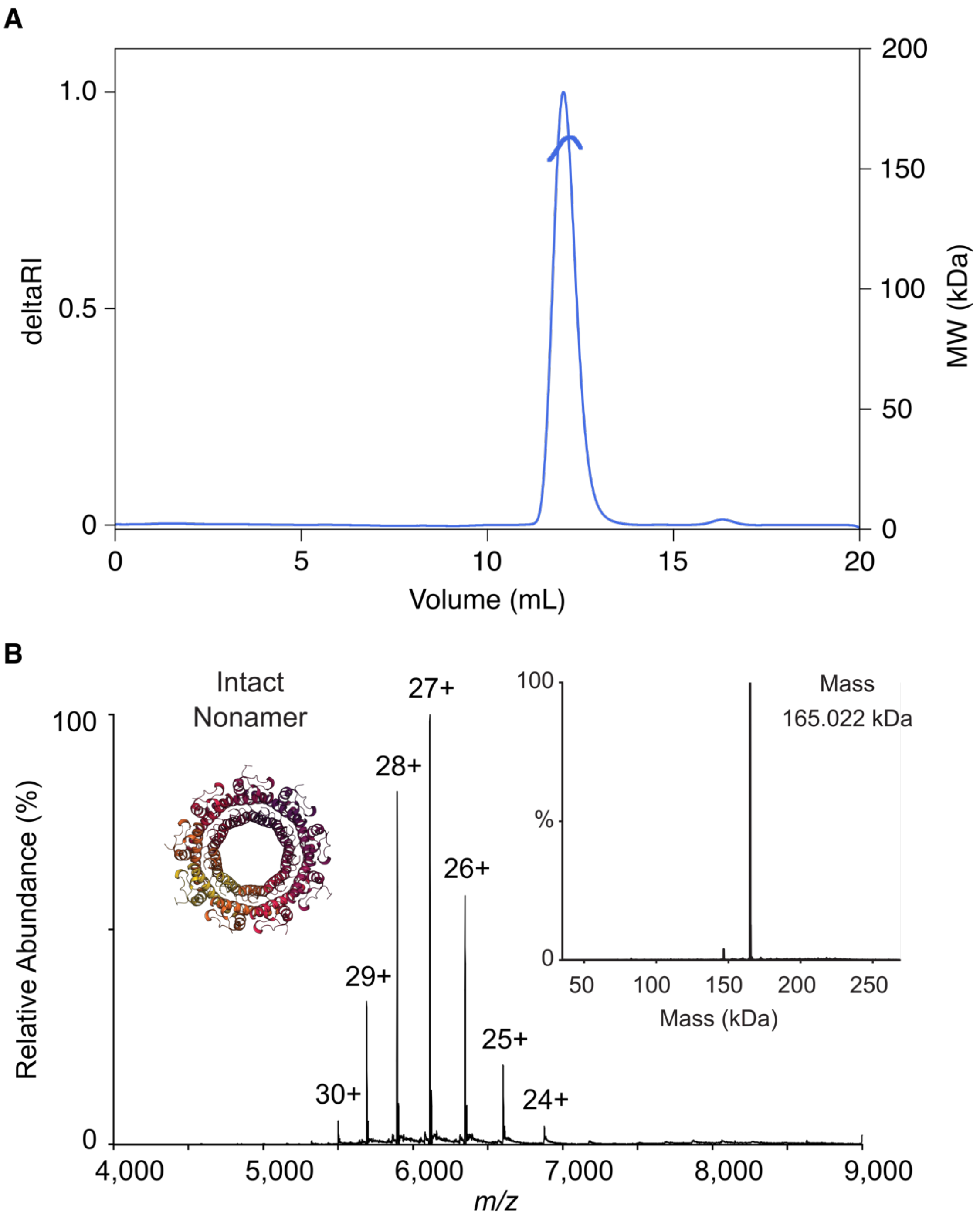

Figure S5. Molecular weight determination of TerS. (**A**) Size-exclusion chromatography and multi-angle laser-light scattering analysis (100 µL 2.7 mg/mL) in 20 mM HEPES, 300 mM KCl, 1 mM DTT, pH 7.0. Protein eluting from a Superdex 200 10/300 GL column (GE Healthcare) was analyzed using an on-line Dawn HELEOS-II light scattering detector and Optilab rEX refractometer (Wyatt). Data were processed based on a refractive index increment of 0.183 mL/g in ASTRA 5.3.4 (Wyatt). (**B**) High resolution Orbitrap native MS measurement showed a single charge state distribution corresponding to a species with a mass of 165.020±0.005 kDa (based on four most intense charge states). The fully deconvoluted spectrum indicated a single species with mass of 165.022 kDa (inset), unambiguously indicating a nonameric assembly based on the known monomer mass of 18.3 kDa.

Table S2. TerS crystallographic data collection and refinement statistics.

	500 mM KI soak	250 mM KI soak
**Data collection**		
Wavelength (Å)	1.6984	0.9763
Space group	*H*3	*H*3
Cell dimensions		
a, *c* (Å)	123.3, 79.4	123.3, 79.0
Resolution (Å)	44.31–1.79 (1.83–1.79)	44.23–1.40 (1.43–1.40)
R_{merge}	0.080 (0.539)	0.049 (0.707)
$I / \sigma(I)$	15.2 (1.4)	10.3 (1.1)
$CC_{1/2}$ (%)	99.6 (51.5)	99.7 (52.5)
Completeness (%)	95.3 (47.3)	98.8 (88.6)
Redundancy	4.4 (1.2)	2.7 (2.0)
Wilson B (Å^2)	19.0	18.6
No. unique reflections	40412 (1184)	86871 (3870)
**Refinement**		
Resolution (Å)		44.23–1.40
No. reflections		
Working		82402
Free		4469
R_{work}/R_{free}		0.159/0.170
No. atoms		
Protein		2474
Water		338
Ligand		9
B factors (Å^2)		
Protein		21.9
Water		33.0
Ligand		30.9
R.m.s. deviations		
Bond lengths (Å)		0.011
Bond angles (°)		1.4
Ramachandran		
Favored (%)		100.0
Outlier (%)		0.0

Table S3. Intramolecular contacts in the N-terminal helix-turn-helix-like region of HK97 TerS.

Residue 1	Residue 2	Nature of contact
T25	H43	hydrogen bond between sidechains
I26	W42	hydrogen bond between backbone oxygen and sidechain
P28	W42	CH/π stacking interaction
P29	A32	hydrogen bond between backbone atoms
S30	G33	hydrogen bond between backbone atoms

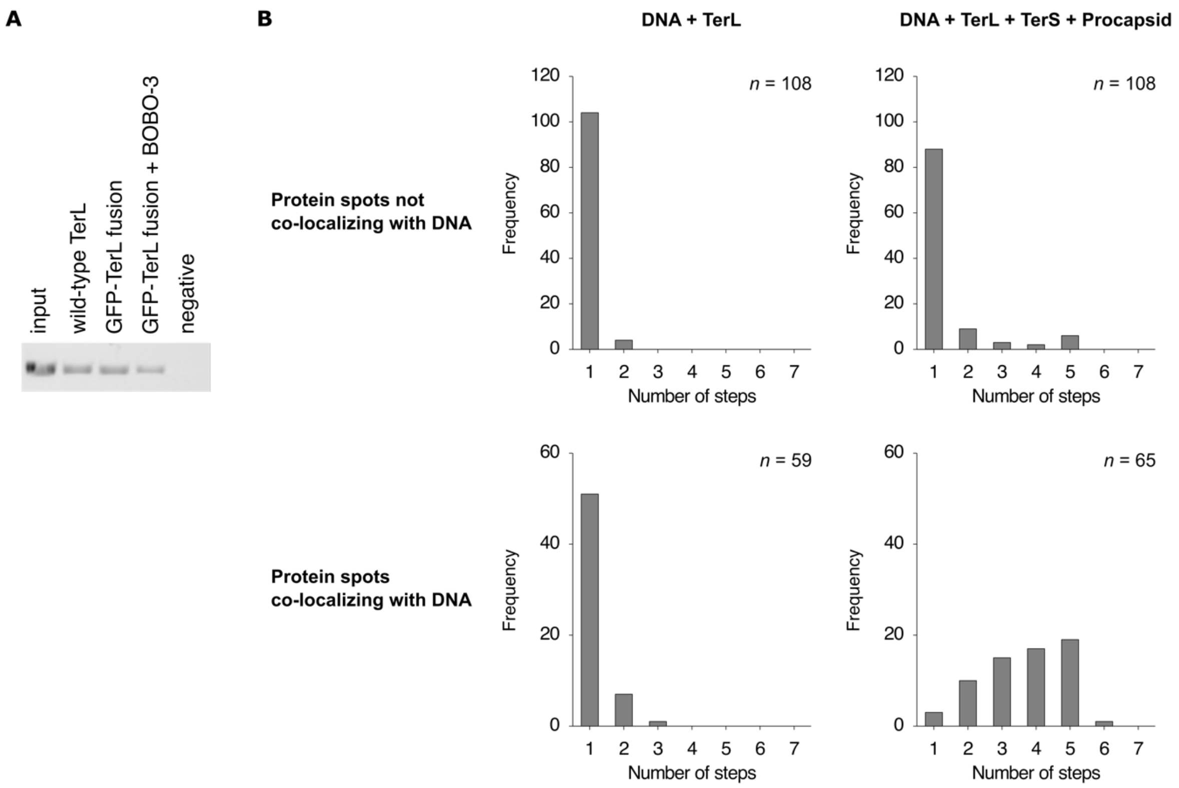

Figure S6. Control packaging experiment and photobleaching step analyses for GFP-TerL. (**A**) Packaging of *Sca*I-linearized *cos*-containing pUC18 DNA by GFP-TerL with and without BOBO-3 DNA staining. A reduction in band intensity was observed with BOBO-3 bound to DNA because the dye interfered with ethidium bromide staining (data not shown). (**B**) GFP-TerL photobleaching steps observed by total internal reflection fluorescence microscopy. Left column, GFP-TerL in the presence of only DNA; right column, GFP-TerL in the presence of DNA, TerS and procapsids. Top row, events not coincident with BOBO3 DNA staining. Bottom row, events coincident with DNA staining.

Table S4. Event counts for the step-wise photobleaching measurements.

	DNA + TerL	DNA + TerL + TerS + Procapsid
**Number of DNA spots**	**699**	**367**
with no co-localizing protein signal	634	259
with co-localizing protein signal, discarded	6	43
with co-localizing protein signal, analyzed	59	65

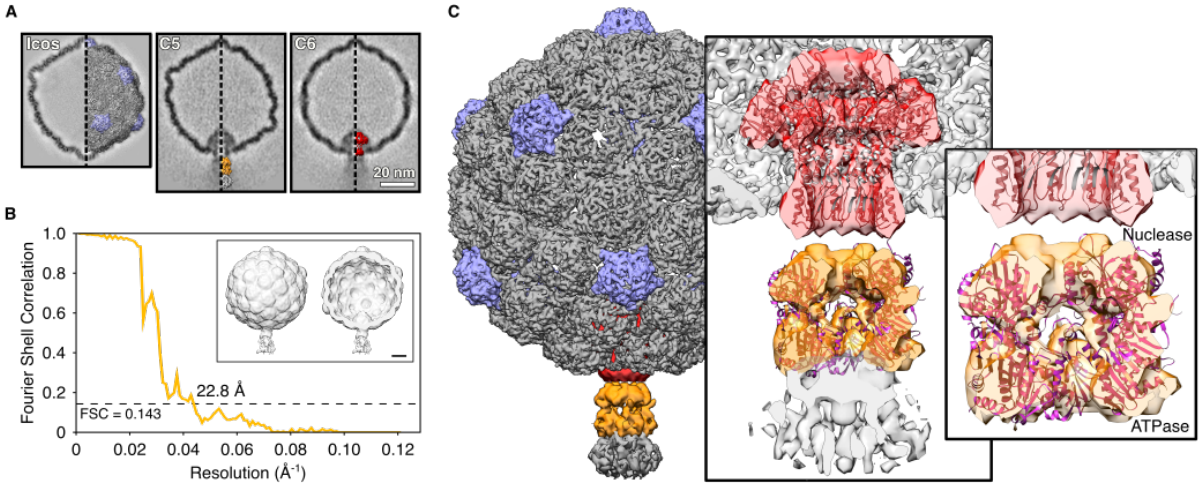

Figure S7. Low-resolution cryo-EM-based model of the HK97 DNA packaging assembly. (**A**) Cross-section of three reconstructions with icosahedral, C5 and C6 symmetry imposed. The poorly resolved densities corresponding to terminases and DNA indicated a dynamic system. 3D densities depicted on the right side of each cross-section were used to generate a composite map. (**B**) Fourier shell correlation analysis of the final half-maps during refinement with no symmetry imposed. Inset shows densities from this C1 refinement (whole and cut-through). Scale bar = 100 Å. (**C**) Composite map showing portal (red) and TerL (orange). Inset, rigid body fitting of a HK97 portal homolog (PDB 3KDR), and model of the HK97 TerL pentamer based on *pac* phage P74-26 pentameric ATPase model. It is unclear if the distal density corresponds to DNA or TerS.

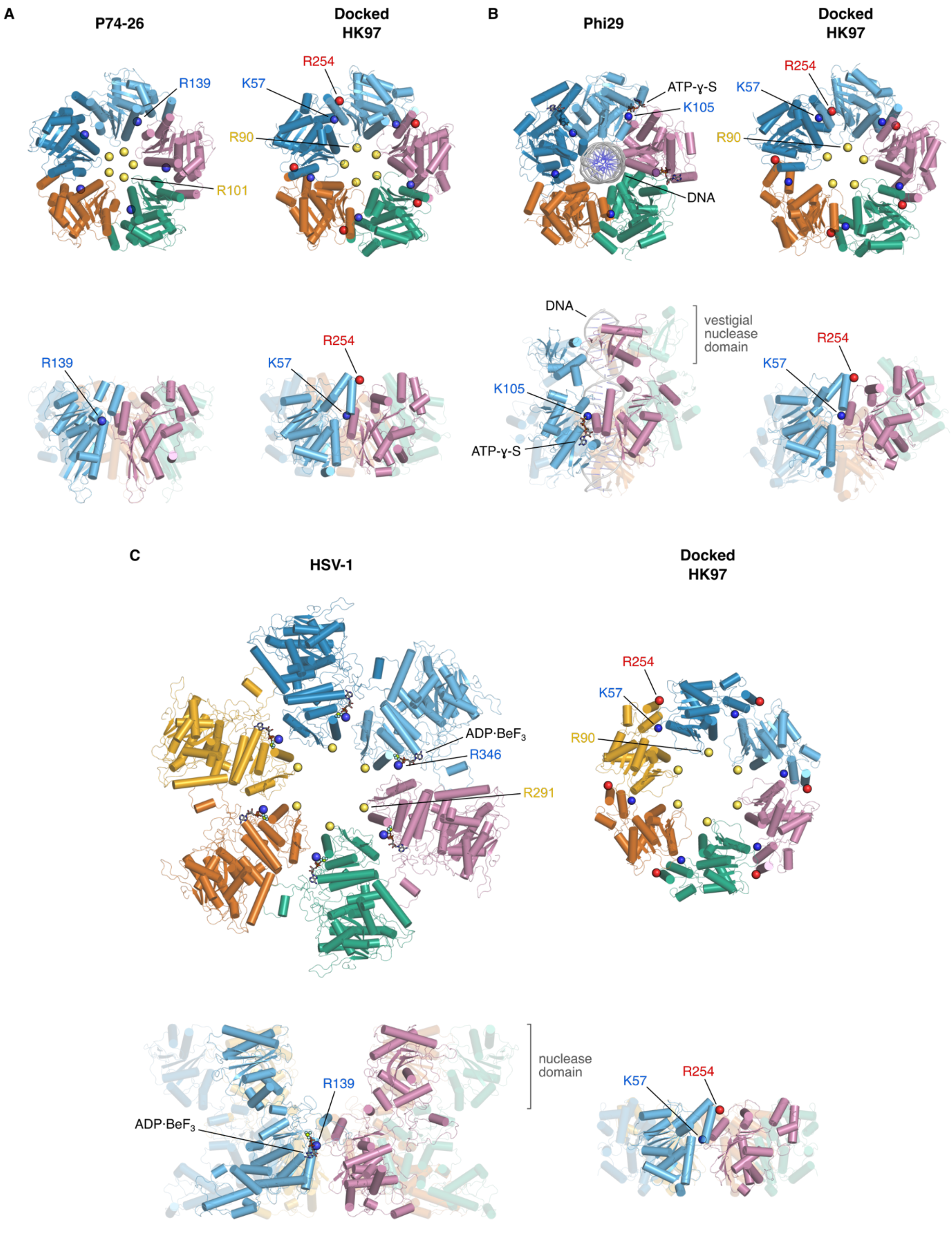

A
P74-26
Docked HK97
R139
R101
K57
R254
R90
B
Phi29
ATP-γ-S
K105
DNA
vestigial nuclease domain
C
HSV-1
ADP·BeF3
R346
R291
nuclease domain

Figure S8. HK97 TerL ATPase domain docked against homologous TerL assemblies. (**A**) Pentameric ATPase model of P74-26 TerL (PDB 4ZNK), generated using M-ZDOCK (3) as in (4). (**B**) Cryo-EM structure of the phi29 DNA packaging assembly (PDB 7JQQ). pRNA was omitted from the cartoon representation for clarity. (**C**) Hexameric terminase assembly of herpes simplex virus type 1 (HSV-1; PDB 6M5V). The identified *trans*-acting arginine finger for each assembly is indicated with a blue sphere. Confirmed or putative DNA-binding residues are marked with a yellow sphere for the P74-26 and HSV-1 assemblies. A superposition of the HK97 TerL ATPase domain is shown on the right, with residues K57, R254 and R90 indicated with a blue, red and yellow sphere, respectively.

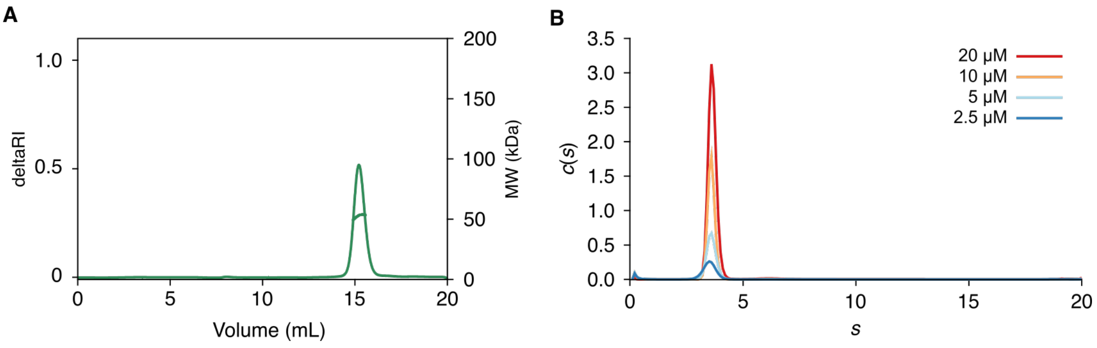

Figure S9. Molecular weight determination of TerL. (**A**) Size-exclusion chromatography and multi-angle laser-light scattering analysis (100 μL 1.2 mg/mL) in 20 mM HEPES, 300 mM KCl, 1 mM DTT, pH 7.0 using a Superdex 200 10/300 GL column (GE Healthcare). (**B**) Sedimentation coefficient distribution by sedimentation velocity analytical ultracentrifugation. Epon double-sector centerpieces were filled with protein (2.5-20 uM in 20 mM HEPES, 300 mM KCl, 1 mM DTT, pH 7.0) and buffer, respectively, and centrifuged in an An-60 Ti rotor at 50000 rpm, 20 °C, for 7.5 h using a Beckman Optima XL/I ultracentrifuge. Absorbance scans were acquired at 280 nm at 7.5 min intervals. A protein partial specific volume of 0.73747 mL/g, buffer density and viscosity of 1.01390 g/mL and 0.01002 P was estimated using Sednterp (5). Data were analyzed under the continuous c(*s*) distribution model using Sedfit (6). Frictional ratio estimates of 1.32–1.37 and standard sedimentation coefficients of 3.66–3.81 S were obtained, contributing to over 90% of the signal.

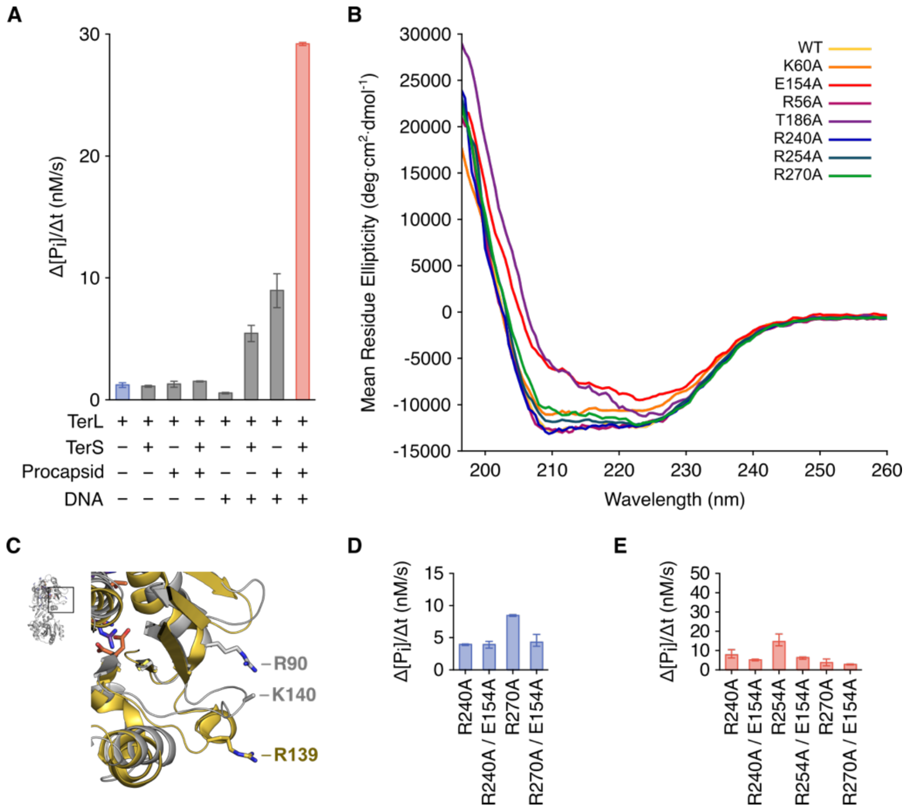

Figure S10. Alanine mutagenesis analysis of TerL. (A) ATP hydrolysis rates at 1 mM ATP concentration, based on inorganic phosphate release, of TerL with different combinations of TerS, procapsid and DNA. The mean and range of three initiate rate measurements are reported for each condition. (**B**) Far-UV circular dichroism spectra of wild-type and mutant HK97 proteins. Spectra were recorded for protein (2–5 μM) in 20 mM Tris sulfate, 50 mM Na_2SO_4, 10 mM $MgSO_4$, 0.5 mM tris(2-carboxyethyl)phosphine hydrochloride, pH 7.5, at 20 °C. (**C**) Superposition of the HK97 TerL structure with the arginine finger of *pac* phage P74-26 TerL (gold, PDB 4ZNK). (**D**, **E**) ATPase rescue experiments conducted with TerL Walker B E154A mutant in equimolar amounts at 1 mM ATP concentration in the absence and presence of DNA, TerS and procapsids, respectively. The mean and range of three initiate rate measurements are reported for each condition.

Table S5. DNA oligonucleotides for cloning and mutagenesis. Primers for plasmid linearization for restriction-free cloning are listed. Numbering on *cos* DNA primers indicates the genome position of the first complementary nucleotide. All sequences are provided in 5' to 3' direction.

**Restriction-free cloning of His-SUMO-fusion**	
SUMO_GP1_for	gaacagattggtggtgcagataaacgaatccgttccg
SUMO_GP1_ rev	tacctaagcttgtctttatccgtgcttgggaaaggc
SUMO_GP2_ for	gaacagattggtggtatgacgcgaggtgagcg
SUMO_GP2_rev	tacctaagcttgtcttcacatgctcagcggtcg
Plasmid_for	agacaagcttaggtatttattcgg
Plasmid_rev	accaccaatctgttctctgtga
**Restriction-free cloning of His-fusion**	
His_GP2_for	atcaccaccaccacatgacgcgaggtgagcg
His_GP2_rev	tgaggagaaggcgcgtcacatgctcagcggtcg
Plasmid_for	cgcgccttctcctcactgttccaggggccccat
Plasmid_rev	tgtggtggtggtgatgatggctgctgccc
**Site-directed mutagenesis**	
GP2_R3A_for	cggcaggtgagcgtgtaatagcgttcatt
GP2_R3A_rev	ctcacctgccgtcatgtggtggtgg
GP2_R6A_for	gtgaggccgtaatagcgttcattgagcg
GP2_R6A_rev	tattacggcctcacctcgcgtcatgtgg
GP2_R13A_for	attgaggccttttgcatcgtgccagaag
GP2_R13A_rev	caaaaggcctcaatgaacgctattacacgc
GP2_R28A_for	ctatggcgttggaccccttcagaaagattt
GP2_R28A_rev	tccaacgccataggttggccgataagc
GP2_R56A_for	cgccgcgaaaaatggtaagactggcc
GP2_R56A_rev	tttttcgcggcgatggagaggatcgc
GP2_R90A_for	gcgcggaacaggcggccatcg
GP2_R90A_rev	tgttccgcgctgagtgcaccgct
GP2_R159A_for	ggttgcgggcccgcaggatgattttatc
GP2_R159A_rev	gcccgcaacctgccctgtttcatcg
GP2_R226A_for	agtaaagccgagtcctggctggctgc
GP2_R226A_rev	gactcggctttactgatatcagcgtcttttgg
GP2_R240A_for	cattcgcgtcagaaaaagacatggcgc
GP2_R240A_rev	ctgacgcgaatgttcccagtgccg
GP2_R247A_for	gcggcccaggctgagaaagctgg
GP2_R247A_rev	ctgggccgccatgtctttttctgacc
GP2_R254A_for	ctggcgcaatgccaagcttcgaaaacac
GP2_R254A_rev	cattgcgccagctttctcagcctggc
GP2_R263A_for	cttcgcgaacctcaacctcaatcagcg
GP2_R263A_rev	aggttcgcgaaggtgttttcgaagcttg
GP2_R270A_for	tcaggccgtgtctaccgtatcgccg
GP2_R270A_rev	acacggcctgattgaggttgaggtttcg
GP2_K140A_for	aaggtgcgacgacgcacggcc
GP2_K140A_rev	cgtcgcaccttctgcggataaagcc
GP2_K204A_for	gtcgcgtcgaaagatccgcacatcg

GP2_K204A_rev	ttcgacgcgaccgcatcatcaatccag
GP2_K206A_for	aatcggccgatccgcacatcgtgtg
GP2_K206A_rev	gatcggccgatttgaccgcatcatcaa
GP2_T186A_for	cagtgcgcaggcagcaaacgatgctga
GP2_T186A_rev	cctgcgcactgataacgattagcagcgg
GP2_K60A_for	ggtgcgactggcctgattgccggaat
GP2_K60A_rev	ccagtcgcaccattttttcgggcgatg
GP2_E154A_for	tcgatgcgacagggcaggttagggg
GP2_E154A_rev	ctgtcgcatcgagaatggccagaatgg
**GFP and linker insertion**	
GFP_for	atcaccaccaccacatggtgagcaagggc
GFP_rev	ccagatccgccgctgccacccttgtacagctcgtccat
Plasmid_for	cagcggcggatctggcggttccggtatgacgcgaggtgag
Plasmid_rev	tgtggtggtggtgatgatggctgctgccc
**Overlap-extension and restriction cloning of HK97 *cos* region**	
cos-312BamHIfor	gtggatccgacggtaagtttgttcagc
cos1strev	caaactttggcggcggtcatttg
cos2ndfor	gaccgccgccaaagtttgaatttaac
cos+472EcoRIrev	gtgaattcccgtgcatcacgatcg
**Generation of 5′ biotinylated DNA**	
cos-80for	atcaaatgagaatgaatcgcatc
cos+150rev	aaacctgcatgggacg-BIO

SUPPLMENTARY NOTE

Analysis of photobleaching events

DNA particles were identified by extracting connected components from an average of the first 500 frames in the red channel. The intensity-weighted centroids of these components were taken to be DNA coordinates. Coordinates fewer than 10 px apart were rejected. For each coordinate in the red channel, given the translation vector from calibrating with TetraSpeck microspheres (ThermoFisher) emitting in both channels, the corresponding pixel in the green channel was found. A circle 3.5 px in radius, large enough only to enclose an event, was drawn around the center of this pixel. Mean intensity of the circle as a function of time was extracted and analyzed for photobleaching steps using the PIF algorithm (7) with Chung-Kennedy filtering (8). Over-fitted steps were identified by generating a counter-fit with steps between the original steps and comparing chi-squared statistics, as introduced by Kerssemakers et al. (9), and subsequently rejected.

For more robust analysis, a moving two-sample t-test (10) and a chi-squared minimization-based step-finding algorithm (9) were also applied. However, this resulted in a systematic under-fitting or over-fitting of steps, owing to the low signal-to-noise ratio of the experiment.

To build a distribution of the number of photobleaching steps per protein event co-localizing with DNA, an event was accepted only if steps could be fitted and if it had an intensity-weighted centroid within experimental error of the centroid of the DNA particle. A centroid-based measure was used because the density of events in the green channel was high and in turn background pixels surrounding each event could not be reliably defined for the fitting of Gaussian distributions. Adapting from Gelles et al. (11), experimental error was estimated to be the precision (two standard deviations) with which the x- and y-position of one GFP-LT monomer could be assigned relative to another, in time. We calculated one standard deviation to be 40.4 nm in both directions.

We note that the number of photobleaching events could be underestimated due to the presence of a dark GFP population as a result of misfolding or photobleaching prior to the experiment; and due to mis-fitting of two photobleaching events as one because the two events occurred within the same video frame, as described in detail by Ulbrich and Isacoff (12). The observed photobleaching rate of the GFP fusion protein was 0.10 s^{-1}, typical of GFP proteins (13). We also acknowledge the possibility of overestimating the number of photobleaching steps when the event monitored was coincident with other passively absorbed GFP molecules.

If one assumed all molecules observed in the DNA-bound motor experiment were pentamers, a binomial distribution with 72% probability of GFP being fluorescent could be fitted. However, it was likely that lower-order species as observed in the absence of prohead and small terminase also contributed to the distribution. Given the complexity of the experiment, it was reasonable to conclude only that the upper limit for the oligomeric state of TerL in a motor assembly was 5.

REFERENCES

1. Duffy,C. and Feiss,M. (2002) The large subunit of bacteriophage λ's terminase plays a role in DNA translocation and packaging termination. *J. Mol. Biol.*, **316**, 547–561.
2. Hang,J.Q., Tack,B.F. and Feiss,M. (2000) ATPase center of bacteriophage λ terminase involved in post-cleavage stages of DNA packaging: identification of ATP-interactive amino acids1. *J. Mol. Biol.*, **302**, 777–795.
3. Pierce,B., Tong,W. and Weng,Z. (2005) M-ZDOCK: a grid-based approach for Cn symmetric multimer docking. *Bioinformatics*, **21**, 1472–8.
4. Hilbert,B.J., Hayes,J.A., Stone,N.P., Duffy,C.M., Sankaran,B. and Kelch,B.A. (2015) Structure and mechanism of the ATPase that powers viral genome packaging. *Proc. Natl. Acad. Sci.*, **112**, E3792-9.
5. Laue,T.M., Shah,B.D., Ridgeway,T.M. and Pelletier,S.L. (1992) Computer-aided interpretation of analytical sedimentation data for proteins. In Harding,S.E., Rowe,A.J., Horton,J.C. (eds), *Analytical ultracentrifugation in biochemistry and polymer science*. The Royal Society of Chemistry, Cambridge, UK, pp. 90–125.
6. Schuck,P. (2000) Size-distribution analysis of macromolecules by sedimentation velocity ultracentrifugation and Lamm equation modeling. *Biophys. J.*, **78**, 1606–1619.
7. McGuire,H., Aurousseau,M.R.P., Bowie,D., Bluncks,R. and Blunck,R. (2012) Automating single subunit counting of membrane proteins in mammalian cells. *J. Biol. Chem.*, **287**, 35912–21.
8. Chung,S.H. and Kennedy,R.A. (1991) Forward-backward non-linear filtering technique for extracting small biological signals from noise. *J. Neurosci. Methods*, **40**, 71–86.
9. Kerssemakers,J.W.J., Laura Munteanu,E., Laan,L., Noetzel,T.L., Janson,M.E. and Dogterom,M. (2006) Assembly dynamics of microtubules at molecular resolution. *Nature*, **442**, 709–712.
10. Carter,N.J. and Cross,R.A. (2005) Mechanics of the kinesin step. *Nature*, **435**, 308–312.
11. Gelles,J., Schnapp,B.J. and Sheetz,M.P. (1988) Tracking kinesin-driven movements with nanometre-scale precision. *Nature*, **331**, 450–453.
12. Ulbrich,M.H. and Isacoff,E.Y. (2007) Subunit counting in membrane-bound proteins. *Nat. Methods*, **4**, 319–321.
13. Pierce,D.W., Hom-Booher,N. and Vale,R.D. (1997) Imaging individual green fluorescent proteins. *Nature*, **388**, 338–338.
